# Bounds on the Transmit Power of *b*-Modulated NFDM Systems in Anomalous Dispersion Fiber

**DOI:** 10.3390/e22060639

**Published:** 2020-06-09

**Authors:** Shrinivas Chimmalgi, Sander Wahls

**Affiliations:** Delft Center for Systems and Control, Delft University of Technology, Mekelweg 2, 2628 CD Delft, The Netherlands; s.wahls@tudelft.nl

**Keywords:** nonlinear Fourier transform, nonlinear frequency division multiplexing, b-modulation, power limitation

## Abstract

The performance of various nonlinear frequency division multiplexed (NFDM) fiber-optic transmission systems has been observed to decrease with increasing signal duration. For a class of NFDM systems known as *b*-modulators, we show that the nonlinear bandwidth, signal duration, and power are coupled when singularities in the nonlinear spectrum are avoided. When the nonlinear bandwidth is fixed, the coupling results in an upper bound on the transmit power that decreases with increasing signal duration. Signal-to-noise ratios are consequently expected to decrease, which can help explain drops in performance observed in practice. Furthermore, we show that there is often a finite bound on the transmit power of *b*-modulators even if spectral singularities are allowed.

## 1. Introduction

The nonlinear Fourier transform (NFT) [1] is a mathematical tool to solve the normalized nonlinear Schrödinger equation (NSE)

(1) $$i\frac{\partial q}{\partial z}+\frac{1}{2}\frac{\partial^{2}q}{\partial t^{2}}+\kappa {\left|q\right|}^{2}q=0,\;q=q\left(z,t\right),$$

which is a model for an ideal lossless single-mode fiber obtained after suitable normalization and path averaging [2]. (The path average can be avoided by using tapered fibers [3].) Here, q(z,t) is the slowly varying pulse envelope, *z* is the location, and *t* is retarded time, all in normalized units. The parameter κ determines if the dispersion in the fiber is normal (−1) or anomalous (+1). The nonlinear evolution of the signal according to the NSE equals a simple phase rotation in the nonlinear Fourier domain (NFD) [1]. Hence, it was suggested to embed data in the NFD at the transmitter and use the NFT to recover the data at the receiver [4,5]. This idea is known as nonlinear frequency division multiplexing (NFDM).

NFDM has garnered much attention in recent years and many different NFDM system designs have been proposed [3,6,7,8,9,10,11,12,13,14,15,16,17]. A common problem with many NFDM designs is that the optimum transmit power decreases with signal duration, making it difficult to utilize signals significantly longer than the channel memory [10,11,18,19,20]. Thus, signals are typically short with a relatively large portion acting as a guard interval that contains no information, leading to low spectral efficiencies. The difficulties with transmitting longer signals have been suspected to be caused by limitations of numerical NFT algorithms and increased signal-noise interactions [18] [p. 3], [11] [Section 3.3], [12] [Section 4].

Recently, in [19], we discovered a new factor contributing to this phenomenon when we derived an upper bound on the transmit power of one specific NFDM system proposed in [21]. It was shown that the transmit power bound decreases with signal duration when the nonlinear bandwidth is kept constant. Since signals with lower power are more susceptible to corruption by noise, this leads to reduced transmission performance. In this paper, we look at a class of systems where only a part of the nonlinear Fourier spectrum known as continuous spectrum is modulated, which is the nonlinear analogue of linear frequency division multiplexing. More specifically, we look at so-called *b*-modulators in the case of anomalous dispersion. The paper is organized as follows. In Section 2, we briefly review nonlinear frequency division multiplexing (NFDM). In Section 3, we derive two different upper bounds on the transmit power of *b*-modulated systems. We conclude our findings in Section 4.

### Notation

Real numbers: R; $R_{\geq 0}\;:=\;\left\{x\in R:x\geq 0\right\}$; Complex numbers: C; Complex numbers with positive imaginary part: H; Integers: Z; Natural numbers: N; $i:=\sqrt{-1}$; Euler’s number: e; Real part: ℜ(·); Imaginary part: ℑ(·); Complex conjugate: ${\left(\cdot \right)}^{*}$; Absolute value: |·|; Lebesque spaces: $L^{p}\left(X\right)$ contains all measurable complex-valued functions *f* on X for which

(2) $${∥f∥}_{p}:=\left\{\begin{array}{cc} (\int_{X}{\left|f(x)\right|}^{p}{dx)}^{1/p}, & \mathrm{if}\;1\leq p<\infty \\ \underset{x\in X}{\sup}\left|f\left(x\right)\right|, & \mathrm{if}\;p=\infty \end{array}\right.<\infty .$$

## 2. Review of NFDM

In this section, we describe the mathematical machinery behind the nonlinear Fourier transform (NFT) and review the idea of nonlinear frequency domain multiplexing (NFDM).

### 2.1. Nonlinear Fourier Transform for Vanishing Signals

The nonlinear Fourier transform (NFT) that solves the NSE (1) is due to Zakharov and Shabat [1]. It transforms any signal q(t) that vanishes sufficiently quickly for t→±∞ from the time domain to the nonlinear Fourier domain through the analysis of the linear ordinary differential equation (ODE)

(3) $$\frac{\partial V(t,\lambda )}{\partial t}=C\left(t,\lambda \right)V\left(t,\lambda \right)=\left[\begin{array}{cc} -i\lambda & q(t)\\ -\kappa q^{*}\left(t\right) & i\lambda \end{array}\right]V\left(t,\lambda \right).$$

The term λ∈C is a spectral parameter similar to the parameter *s* in the Laplace domain. Since |q(t)|→0 fast for t→±∞, the ODE has solutions that fulfill the boundary conditions

(4) $$\begin{array}{cc} V(t,\lambda )= & \left[\phi \left(t,\lambda \right)\;\bar{\phi }\left(t,\lambda \right)\right]\to \left[\begin{array}{cc} e^{-i\lambda t} & 0\\ 0 & -e^{i\lambda t} \end{array}\right]\mathrm{as}\;t\to -\infty ,\\ V(t,\lambda )= & \left[\bar{\psi }\left(t,\lambda \right)\;\psi \left(t,\lambda \right)\right]\to \left[\begin{array}{cc} e^{-i\lambda t} & 0\\ 0 & e^{i\lambda t} \end{array}\right]\mathrm{as}\;t\to \infty . \end{array}$$

The matrix V(t,λ) is said to contain (generalized) eigenfunctions since Equation (3) can be rearranged into an eigenvalue equation with respect to λ [22]. For the solutions Equation (4) of Equation (3), there exists a unique matrix

(5) $$S\left(\lambda \right)=\left[\begin{array}{cc} a(\lambda ) & \bar{b}\left(\lambda \right)\\ b(\lambda ) & -\bar{a}\left(\lambda \right) \end{array}\right],$$

called the scattering matrix, such that [22]

(6) $$\left[\begin{array}{cc} \phi (t,\lambda ) & \bar{\phi }\left(t,\lambda \right) \end{array}\right]=\left[\begin{array}{cc} \bar{\psi }\left(t,\lambda \right) & \psi (t,\lambda ) \end{array}\right]S\left(\lambda \right).$$

The components a(λ), b(λ), $\bar{b}\left(\lambda \right)$, and $\bar{a}\left(\lambda \right)$ are known as the *scattering coefficients*. The scattering coefficients satisfy [22] (pp. 260, 271)

(7) $$\bar{b}\left(\lambda \right)=\kappa b^{*}\left(\lambda^{*}\right),\;\bar{a}\left(\lambda \right)=a^{*}\left(\lambda^{*}\right),\;a\left(\lambda \right)\bar{a}\left(\lambda \right)+b\left(\lambda \right)\bar{b}\left(\lambda \right)=1.$$

The evolution of the scattering coefficients along the location *z* in the fiber is simple: [22] [Sec. III]

(8) $$\begin{array}{cc} a(z,\lambda ) & =a(0,\lambda ),\\ b(z,\lambda ) & =b\left(0,\lambda \right)e^{-4i\lambda^{2}z}. \end{array}$$

The *reflection coefficient* is then defined as ρ(λ)=b(λ)/a(λ) for λ∈R. It provides a represention of the *continuous spectrum*. In the anomalous dispersion case κ=1 considered in this paper, the nonlinear Fourier spectrum can also contain a so-called *discrete spectrum*. It corresponds to the complex poles of the reflection coefficient in the upper half-plane H, or equivalently to the zeros $\lambda_{k}\in H$ of a(λ). Usually, there are only finitely many (N) such poles, all simple [22] [Section VI]. The poles $\lambda_{k}$ are also referred to as *eigenvalues* and a corresponding set of values $\rho_{k}:=b\left(\lambda_{k}\right)/\frac{da}{d\lambda }\left(\lambda_{k}\right)$ are known as residues [22] [App. 5]. The eigenvalues correspond to the solitonic components of the signal. There are different ways to define a nonlinear Fourier spectrum. One possibility is ${\left\{\rho \left(\lambda \right)\right\}}_{\lambda \in R}$, ${\left(\lambda_{k},\rho_{k}\right)}_{k=1}^{N}$ [22]. Another is ${\left\{b\left(\lambda \right)\right\}}_{\lambda \in R}$, ${\left(\lambda_{k},b\left(\lambda_{k}\right)\right)}_{k=1}^{N}$ [23]. In the case of anomalous dispersion (κ=1), the energy of the signal q(t) is related to the components of the nonlinear spectrum as [4] [p. 9]

(9) $$\int_{-\infty }^{\infty }{\left|q\left(t\right)\right|}^{2}dt=\frac{1}{\pi }\int_{-\infty }^{\infty }{\log(1+|\rho \left(\xi \right)|}^{2})d\xi +4\sum_{k=1}^{N}ℑ\left(\lambda_{k}\right),$$

or equivalently

(10) $$\int_{-\infty }^{\infty }{\left|q\left(t\right)\right|}^{2}dt=-\frac{1}{\pi }\int_{-\infty }^{\infty }{\log(1-|b\left(\xi \right)|}^{2})d\xi +4\sum_{k=1}^{N}ℑ\left(\lambda_{k}\right).$$

Note that Equation (7) implies that |b(ξ)|≤1 for real ξ. When |b(ξ)|=1 for some real ξ, then the integrand in Equation (10) is undefined at that point. Such points are known as *spectral singularities* in the literature [24]. Even though simple signals such as the rectangle and hyperbolic secant can have isolated spectral singularities [25] [Chapter 2], most of the literature on NFTs assumes that |b(ξ)|<1 for all real ξ. From here on, ξ will be used to denote the spectral parameter if it is strictly real and λ if otherwise.

Information can be embedded in the scattering coefficients in various ways. In this paper, we consider the techniques where information is embedded only in b(ξ) for ξ∈R, i.e., we consider signals without solitons. The idea of embedding information in b(ξ) is known as *b*-modulation [26]. The advantages of *b*-modulation are tight control over signal duration and lower sensitivity w.r.t. noise [11,26]. If the signals are of infinite duration, they are truncated to some finite interval $\left[T_{1},T_{2}\right]$. From Equation (10), we can see that the energy of a *b*-modulated signal can be controlled by varying b(ξ). This indirectly allows us to control the average power of the truncated signal. In this paper, we will concentrate on *b*-modulation in the case of anomalous dispersion (κ=1).

### 2.2. NFDM Signal Generation

**Figure 1:**
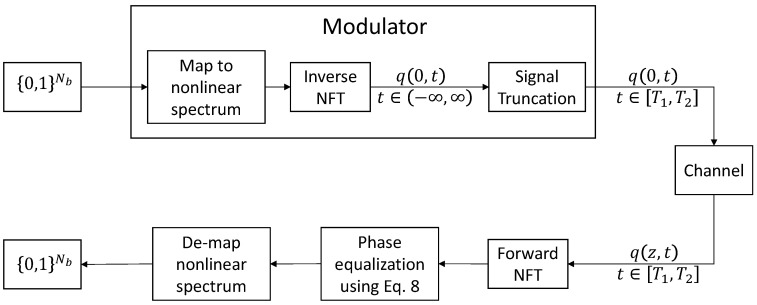
Nonlinear frequency domain multiplexing (NFDM) transmission of one block of $N_{b}$ bits.

As in any digital transmission scheme, the data to be transmitted are fed to the transmitter as a stream of bits. The system then takes a block of $N_{b}\in N$ bits and generates a signal for transmission through the optical fiber channel. This is the process of modulation. At the receiver, the effect of the channel on the nonlinear spectrum is first reverted using Equation (8). Then, the block of bits is recovered. The NFDM transmission scheme is illustrated in Figure 1.

**Figure 2:**
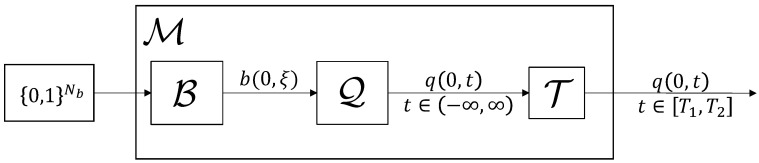
Transmitter side components of a NFDM transmission scheme employing b-modulation.

In order to be able to make concise statements in the coming sections, we now introduce formal definitions for a modulator and a *b*-modulator. An illustrating block diagram is shown in Figure 2.

**Definition** **1.** *A* modulator *is a function-valued function*

(11) $$M:{\left\{0,1\right\}}^{N_{b}}\to L^{2}\left(\left[T_{1},T_{2}\right]\right)$$

*that maps vectors of $N_{b}$ bits to transmit signals of finite energy and duration.*

This definition of a modulator makes no assumptions about how data are embedded in the signal q(t) and is thus very general. A *b*-modulator on the other hand is a specific type of modulator that embeds data in the scattering coefficient b(ξ) that was defined in Equation (5).

**Definition** **2.** *A b*-modulator* is a modulator of the form*

(12) M(v)=T(Q(B(v))),

*where B maps vectors of bits to nonlinear spectra b(ξ) with ξ∈R, Q is the inverse NFT that maps scattering coefficients b(ξ) to the corresponding time-domain signals q(t), t∈R, without solitonic components, and*

(13) $$T:L^{2}\left(R\right)\to L^{2}\left(\left[T_{1},T_{2}\right]\right),\;\left[T\left(q\right)\right]\left(t\right)=q\left(t\right)\;\forall \;t\in \left[T_{1},T_{2}\right],$$

*simply truncates infinite duration signals to a finite duration. We assume that b=B(v) and q=Q(b) satisfy*

(14) $${∥b∥}_{\infty }\leq 1,\;\int_{-\infty }^{\infty }{\left|q\left(t\right)\right|}^{2}dt=-\frac{1}{\pi }\int_{-\infty }^{\infty }{\log(1-|b\left(\xi \right)|}^{2}{)d\xi <\infty ,\;\forall \;v\in \left\{0,1\right\}}^{N_{b}}.$$

**Remark** **1.** *The first assumption in Equation *(14)* is necessary since ${\left|a\left(\xi \right)\right|}^{2}+{\left|b\left(\xi \right)\right|}^{2}=1$ on the real axis for any NFT (see Equation *(7)*). Note that we do not make the common stronger assumption that ${∥b∥}_{\infty }<1$ (no spectral singularities). The second assumption in Equation *(14)* is simply Equation *(10)* specialized to nonlinear spectra without solitonic components. It is known to hold in the absence of spectral singularities. We expect this result to hold even in the presence of spectral singularities. However, as we could not find this result in the literature, we are stating it as an assumption here. We remark that, even if b-modulators that satisfy Equation *(14)* with ${∥b∥}_{\infty }=1$ would not exist, our results still apply to any b-modulator that ensures ${∥b∥}_{\infty }<1$. This still includes all cases in the current literature.*

**Remark** **2.** *For sufficiently rapidly decaying b(ξ) with ${∥b∥}_{\infty }<1$, it is possible to verify that the second integral in Equation *(14)* will be finite. However, when ${∥b∥}_{\infty }=1$, the integrand will have singularities at which it becomes infinite. The integral may or may not be infinite in such cases. It was observed in [21] that it remains finite in specific cases. In this paper, we will show in Lemma 1 that this behavior is the norm, not the exception.*

**Remark** **3.** *The results that will be derived in this paper for b-modulated systems also apply to ρ-modulated NFDM systems with normal dispersion (κ=−1, see, e.g., [27,28]) when b is replaced by ρ. Let us check that the assumptions in Equation *(14)* are fulfilled by ρ in that case. For normal dispersion, ${\left|a\left(\xi \right)\right|}^{2}-{\left|b\left(\xi \right)\right|}^{2}=1$ [29] [p. 25]. Thus, ρ=b/a satisfies ${∥\rho ∥}_{\infty }\leq 1$. Using [29] [Equations 1.6.7 and 1.6.21b], the signal energy is found to satisfy*

(15) $$\int_{-\infty }^{\infty }{\left|q\left(t\right)\right|}^{2}dt=-\frac{1}{\pi }\int_{-\infty }^{\infty }{\log(1-|\rho \left(\xi \right)|}^{2})d\xi .$$

In the next section, we will derive two different bounds on the transmit power of information bearing signals q(0,t) that are generated by *b*-modulators at the fiber input.

## 3. Upper Bounds on the Transmit Power of *b*-Modulators

With fiber-optic transmission systems that modulate the conventional “linear” Fourier spectrum, the power of the transmit signal can theoretically be made arbitrarily high without increasing the bandwidth or duration of the signal, simply by scaling (amplifying) it in the time domain. Although *b*-modulated systems are in many ways similar to such linear systems, there are also some important differences. Scaling the signal in time domain distorts its nonlinear Fourier spectrum in complicated ways. For example, scaling a signal without solitonic components can give rise to many solitons. In linear systems, bandwidth and signal duration are coupled, but the transmit power is independent of the two. We show in the following that, under certain conditions the nonlinear bandwidth, duration, and transmit power in *b*-modulators are coupled. We already showed this for one specific *b*-modulator in [19]. In this section, we will derive two more general bounds on the power of *b*-modulated systems that apply to many systems considered in the literature. In Section 3.1, we derive and discuss a bound for systems that have no spectral singularities. In Section 3.2, we will show that, even in the presence of spectral singularities, the power still remains bounded for a class of *b*-modulators.

### 3.1. Power Bound for a Fixed Gap to Singularity

As already discussed earlier, in the case of anomalous dispersion, it is required that |b(ξ)|≤1 for real ξ. In special cases, even simple signals such as the rectangle and hyperbolic secant can have isolated spectral singularities at which |b(ξ)|=1 [25] [Chapter 2]. In the presence of spectral singularities, the usual theory behind the NFT unfortunately breaks down and has be to extended in a quite complicated manner [24]. Many algorithms available in literature for computing the time-domain signal starting from b(ξ) break down in their presence [26,30]. In practice, to avoid the complications arising from the spectral singularities, a “gap to singularity” $\varepsilon_{b}:=1-{∥b∥}_{\infty }^{2}>0$ is typically enforced by either clipping [19,31] (The use of clipping in [31] was reported in [12] [p. 1574], not in the paper itself.), scaling [26,31], and/or reshaping [12] of b(ξ). In [21], the constellation was reshaped. The gap to singularity $\varepsilon_{b}$ cannot be made arbitrarily small as the numerical algorithms are limited by the computing precision. Since any number that is closer to one than the machine precision is rounded to one, gaps to singularity smaller than machine precision cannot be represented with floating point numbers. As soon as the gap to singularity is never zero, the following power bound applies.

**Theorem** **1.** *Let M be any b-modulator (see Definition 2) with a gap to singularity. That is,*

(16) $$\varepsilon :=1-\max_{v\in {\left\{0,1\right\}}^{N_{b}},\;b=B\left(v\right)}{∥b∥}_{\infty }^{2}>0.$$

*Then, the maximum transmit power of the modulator is upper bounded as*

(17) $$P_{\max}=\max_{q=M\left(v\right),\;v\in {\left\{0,1\right\}}^{N_{b}}}\frac{1}{T_{2}-T_{1}}\int_{T_{1}}^{T_{2}}{\left|q\left(t\right)\right|}^{2}dt\leq \frac{-2W\log\varepsilon }{\pi \gamma (T_{2}-T_{1})},$$

*where 0<γ<1 can be chosen arbitrarily and W>0 is any finite constant such that*

(18) $$\gamma E:=-\frac{\gamma }{\pi }\int_{-\infty }^{\infty }{\log(1-|b\left(\xi \right)|}^{2})d\xi \;\leq \;E_{W}:=-\frac{1}{\pi }\int_{-W}^{W}{\log(1-|b\left(\xi \right)|}^{2})d\xi ,\;\forall \;b=B\left(v\right).$$

*It is always possible to find such a W.*

**Figure 3:**
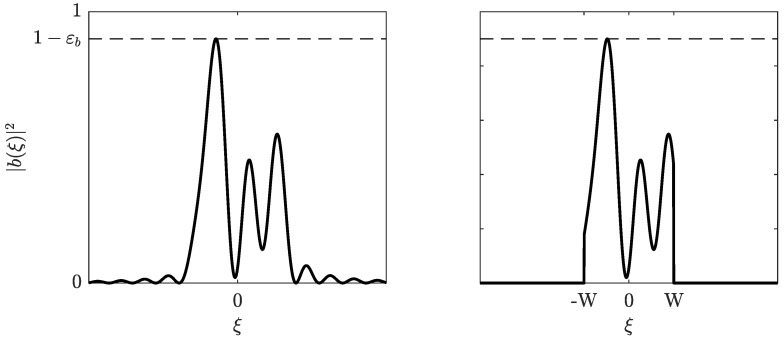
In this example, 2W is exactly the 90% bandwidth: 90% (γ=0.9) of the energy corresponding to the left spectrum (E) are equal to the energy corresponding to right spectrum ($E_{W}$). That is, $\gamma E=E_{W}$.

**Figure 4:**
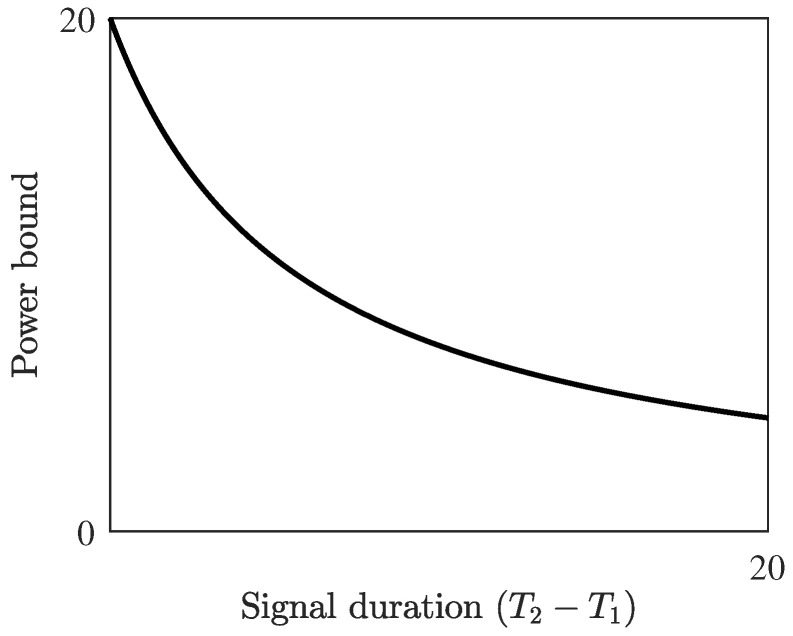
The power bound from Theorem 1 for W=6.0338, γ=0.9 and ε≥0.1. The transmit power of *any* b-modulator with these fundamental parameters must approach zero for long durations.

**Remark** **4.** *Note that 2W is a bound on the nonlinear γ×100-percent bandwidth of the modulator, which is illustrated in Figure 3 together with the gap to singularity $\varepsilon_{b}$. Figure 4 illustrates the decay of the power bound.*

**Remark** **5.** *The most important implication of Theorem 1 is that as soon as there is a nonlinear bandwidth constraint ($\gamma E\leq E_{W}$) and the gap to singularity cannot be made arbitrarily small (e.g., due to clipping or finite precision effects), the transmit power of any b-modulator producing long transmit signals must be low. Longer signals are preferred as they are more data dense. However, making the signals longer decreases the SNRs. Hence, one expects there to be a finite optimal signal duration.*

**Proof of Theorem** **1.** Let 0<γ<1 be fixed. We start with finding W>0. For any fixed b=B(v), the assumptions in Equation (14) ensure that $\gamma E\leq E_{W_{b}}$ for some finite $W_{b}>0$. Since the number of bit vectors $v\in {\left\{0,1\right\}}^{N_{b}}$ that can be passed to the modulator is finite, there is only a finite number of nonlinear spectra b=B(v). Hence, Equation *(18)* is fulfilled if we choose *W* to be the largest of the $W_{b}$.

For any fixed q=M(v) with corresponding b=B(v), the transmit power satisfies

(19) $$\begin{array}{cc} P_{b} & =\frac{1}{T_{2}-T_{1}}\int_{T_{1}}^{T_{2}}{\left|q\left(t\right)\right|}^{2}dt\\ \; & \leq \frac{1}{T_{2}-T_{1}}\underset{=E}{\underset{︸}{\int_{-\infty }^{\infty }{\left|q\left(t\right)\right|}^{2}dt}}\\ \; & \overset{\left(18\right)}{\leq }\frac{1}{T_{2}-T_{1}}\frac{E_{W}}{\gamma }\\ \; & =\frac{1}{T_{2}-T_{1}}\frac{-1}{\pi \gamma }\int_{-W}^{W}{\log(1-|b\left(\xi \right)|}^{2})d\xi \\ \; & \overset{\left(16\right)}{\leq }\frac{1}{T_{2}-T_{1}}\frac{-1}{\pi \gamma }\int_{-W}^{W}\log\left(1-\left(1-\varepsilon \right)\right)d\xi \\ \; & =\frac{1}{T_{2}-T_{1}}\frac{-1}{\pi \gamma }2W\log\varepsilon . \end{array}$$

Since this bound is independent of v, we obtain Equation (17). □

### 3.2. Uniform Power Bound for Arbitrary Gaps to Singularity

The bound derived in Theorem 1 describes many practical situations and applies to most of the *b*-modulators currently seen in the literature. However, the bound is not meaningful in the limit ε→0 as it blows up. It is interesting to know if we could achieve arbitrary powers in scenarios where the gap to singularity could be made arbitrarily small. In the following Theorem 2, we show that the power will still remain bounded for many typical *b*-modulators even in the limit ε→0.

**Theorem** **2.** *Let M be a b-modulator (see Definition 2) such that any b=B(v) is of the form*

(20) $$b\left(\xi \right)=A\sum_{n=-N}^{N}s_{n}\Psi \left(\xi -n\Delta \xi \right),\;s_{-N},\cdots ,s_{N}\in S_{*},\;A,\;\Delta \xi >0,$$

where $S_{*}\subset C$ is a finite constellation alphabet and $\Psi \in L^{2}\left(R\right)$ is a real-analytic carrier waveform with

(21) $$\lim_{\xi \to \pm \infty }\Psi \left(\xi \right)=0\;\mathrm{and}\;\underset{k=2,3,\ldots }{\sup}{∥\frac{d^{k}\Psi }{d\xi^{k}}∥}_{\infty }<\infty .$$

*The power control factor A≥0 and the symbols $s_{n}$ in Equation *(20)* may depend on the bit vector v. All other quantities are assumed independent of v. Then, the maximum transmit power of the modulator is bounded as*

$$P_{\max}=\max_{q=M\left(v\right),\;v\in {\left\{0,1\right\}}^{N_{b}}}\frac{1}{T_{2}-T_{1}}\int_{T_{1}}^{T_{2}}{\left|q\left(t\right)\right|}^{2}dt\leq \frac{\bar{E}}{T_{2}-T_{1}}<\infty ,$$

*where the constant $\bar{E}$ is independent of the power control factors A=A(v) and data symbols $s_{n}=s_{n}\left(v\right)$ in Equation *(20)*, as well as of the temporal domain $\left[T_{1},T_{2}\right]$.*

The proof of Theorem 2 requires us to establish some lemmas first, which will be given later in this section. Before we proceed to the lemmas, let us first discuss the theorem.

Theorem 2 is formulated such that it is applicable to the carriers typically used in NFDM systems. One of the commonly used carriers is the sinc function [10,11,12,32]

(22) $$\Psi \left(\xi \right)=\mathrm{sinc}\left(\xi \right)=\left\{\begin{array}{cc} 1, & \xi =0\\ \frac{\sin(\xi )}{\xi }, & \mathrm{otherwise} \end{array}\right..$$

The function sinc(ξ) is real-analytic [33], square-integrable, and decays to zero as ξ→±∞. To apply Theorem 2, we need to show that $\sup_{k=2,3,\ldots }{∥\frac{d^{k}}{d\xi^{k}}\mathrm{sinc}∥}_{\infty }<\infty$. To check this, we first note that $\mathrm{sinc}\in L^{\infty }\left(R\right)$ with ${\mathrm{sinc}}_{\infty }=1$. The Fourier transform of sinc(ξ) is furthermore a rectangle function,

(23) $$F\left\{\mathrm{sinc}\right\}\left(\omega \right)=\left\{\begin{array}{cc} \pi , & |\omega |<1\\ 0, & \mathrm{otherwise} \end{array}\right..$$

The set of ω for which the Fourier transform is non-zero thus satisfies suppF{sinc}(ω)⊂[−1,1], where “supp” is short for support. Then, [34] [Theorem 4] tells us that ${\frac{d^{k}}{d\xi^{k}}\mathrm{sinc}}_{\infty }\leq 1^{k}{\mathrm{sinc}}_{\infty }$ so that Equation (21) is indeed fulfilled. Theorem 2 now tells us that the *b*-modulator is bounded in transmit power.

**Remark** **6.** *The argument above for showing that the sinc(ξ) carrier satisfies the conditions in Theorem 2 exploits that the support of its Fourier transform is contained in the interval [−1,1]. For a b-modulator M as in Theorem 2 where the Fourier transform is not contained in [−1,1], but in some larger interval [−α,α], the following trick can be applied. For any b=B(v), we define $b_{\alpha }\left(\xi \right)=b\left(\alpha \xi \right)$ and $q_{\alpha }:=Q\left(b_{\alpha }\right)$. By basic properties of the NFT, we have that $q_{\alpha }\left(t\right)=\frac{1}{\alpha }q\left(t/\alpha \right)$. The b-modulator*

(24) $$M_{\alpha }:{\left\{0,1\right\}}^{N_{b}}\to L^{2}\left(\left[\alpha T_{1},\alpha T_{2}\right]\right),\;v\mapsto q_{\alpha }$$

*has the maximum transmit power*

(25) $$P_{\alpha }:=\frac{1}{\alpha T_{2}-\alpha T_{1}}\int_{\alpha T_{1}}^{\alpha T_{2}}|q_{\alpha }{\left(t\right)|}^{2}dt=\frac{1}{\alpha T_{2}-\alpha T_{1}}\int_{\alpha T_{1}}^{\alpha T_{2}}\frac{{\left|q\left(t/\alpha \right)\right|}^{2}}{{\left|\alpha \right|}^{2}}dt=\frac{1}{{\left|\alpha \right|}^{2}\left(T_{2}-T_{1}\right)}\int_{T_{1}}^{T_{2}}{\left|q\left(\tilde{t}\right)\right|}^{2}d\tilde{t}=\frac{P}{{\left|\alpha \right|}^{2}},$$

*where we used the substitution $\tilde{t}:=t/\alpha$, $d\tilde{t}=dt/\alpha$. The carrier waveform of $M_{\alpha }$ will by construction have a Fourier transform with support in [−1,1], so that the argument given above for sinc(ξ) can again be made. Thus, the power P of the modulator M will also be bounded.*

Similar arguments show that raised cosine carriers [26] and flat-top carriers [35] also fulfill the conditions of Theorem 2. Having seen that Theorem 2 is applicable to many *b*-modulated systems, we now prove two lemmas which we need to prove the theorem.

**Lemma** **1.** *Let b(ξ) be any real-analytic function for ξ∈R with*

(26) $${∥b∥}_{\infty }\leq 1,\;\lim_{\xi \to \pm \infty }b\left(\xi \right)=0\;\mathrm{and}\;\underset{k=2,3,\ldots }{\sup}{∥\frac{d^{k}b}{d\xi^{k}}∥}_{\infty }<\infty .$$

*Then, the energy contained in any finite interval [−W,W] is finite:*

(27) $$E_{W}:=-\frac{1}{\pi }\int_{-W}^{W}{\log(1-|b(\xi )|}^{2})d\xi <\infty .$$

**Proof.**  Let us set $f\left(\xi \right)b\left(\xi \right)\bar{b}\left(\xi \right)$, where $\bar{b}\left(\xi \right)=b^{*}\left(\xi^{*}\right)$. If b(ξ) is real-analytic, then $\bar{b}\left(\xi \right)$ is real-analytic which implies f(ξ) is also real-analytic [36] [Proposition 1.1.4]. For ξ∈R, ${f\left(\xi \right)|b(\xi )|}^{2}$. Let $\xi_{0}$ denote any spectral singularity (i.e., $|b(\xi_{0})|=1$). We are interested in showing that the contribution of the singularity to the signal energy is finite, i.e.,

(28) $$I:=\int_{\xi_{0}-\delta /2}^{\xi_{0}+\delta /2}\log\left(1-f\left(\xi \right)\right)d\xi >-\infty$$

for δ>0 small enough. Since f(ξ)∈[0,1], this would imply that *I* is real and not positive. In a interval $\left(\xi_{0}-\delta /2,\xi_{0}+\delta /2\right)$ with δ>0 small enough, we can write ([36] [Corollary 1.1.10])

(29) $$f\left(\xi \right)=f\left(\xi_{0}\right)+\frac{f^{\left(1\right)}\left(\xi_{0}\right)}{1!}\left(\xi -\xi_{0}\right)+\frac{f^{\left(2\right)}\left(\xi_{0}\right)}{2!}{\left(\xi -\xi_{0}\right)}^{2}+\frac{f^{\left(3\right)}\left(\xi_{0}\right)}{3!}{\left(\xi -\xi_{0}\right)}^{3}+\ldots ,$$

where $f^{\left(k\right)}\frac{d^{k}}{d\xi^{k}}f$. The derivative test tells us that $\xi_{0}$ will be an isolated maximum point of *f* only if $f^{\left(k\right)}\left(\xi_{0}\right)=0$ for k=1,…,n with *n* odd and $f^{\left(n+1\right)}\left(\xi_{0}\right)<0$. Plugging these into Equation (29), we get

(30) $$f\left(\xi \right)=1+\frac{f^{\left(n+1\right)}\left(\xi_{0}\right)}{(n+1)!}{\left(\xi -\xi_{0}\right)}^{\left(n+1\right)}+\frac{f^{\left(n+2\right)}\left(\xi_{0}\right)}{(n+2)!}{\left(\xi -\xi_{0}\right)}^{\left(n+2\right)}+\ldots .$$

(Spectral singularities are maximum points because $f\left(\xi \right)={\left|b\left(\xi \right)\right|}^{2}\leq 1$ for all ξ. They must be isolated because otherwise $f\left(\xi \right)={\left|b\left(\xi \right)\right|}^{2}=1$ for all ξ∈R since *f* is real-analytic [36] [Corollay 1.2.6], which contradicts the second condition in Equation (26).)

For showing Equation (28), let us define a second integral II by substituting only the first two non-zero terms of the expansion Equation (30) for f(ξ) in Equation (28):

(31) $$\begin{array}{cc} II & :=\int_{\xi_{0}-\delta /2}^{\xi_{0}+\delta /2}\log\left(1-\left(1+\frac{f^{\left(n+1\right)}\left(\xi_{0}\right)}{(n+1)!}{\left(\xi -\xi_{0}\right)}^{\left(n+1\right)}\right)\right)d\xi \end{array}$$

(32) $$\begin{array}{cc} \; & =\int_{\xi_{0}-\delta /2}^{\xi_{0}+\delta /2}\log\left(-\frac{f^{\left(n+1\right)}\left(\xi_{0}\right)}{(n+1)!}{\left(\xi -\xi_{0}\right)}^{\left(n+1\right)}\right)d\xi \end{array}$$

(33) $$\begin{array}{cc} \; & =\int_{\xi_{0}-\delta /2}^{\xi_{0}+\delta /2}\log\left(-\frac{f^{\left(n+1\right)}\left(\xi_{0}\right)}{(n+1)!}\right)d\xi +\int_{\xi_{0}-\delta /2}^{\xi_{0}+\delta /2}\log\left({\left(\xi -\xi_{0}\right)}^{\left(n+1\right)}\right)d\xi \end{array}$$

(34) $$\begin{array}{cc} \; & =\delta \log\left(-\frac{f^{\left(n+1\right)}\left(\xi_{0}\right)}{(n+1)!}\right)+\int_{-\delta /2}^{\delta /2}\log\left({\left(\xi^{2}\right)}^{(n+1)/2}\right)d\xi \end{array}$$

(35) $$\begin{array}{cc} \; & =\delta \log\left(-\frac{f^{\left(n+1\right)}\left(\xi_{0}\right)}{(n+1)!}\right)+2\frac{n+1}{2}\int_{0}^{\delta /2}\log\left(\xi^{2}\right)d\xi \end{array}$$

(36) $$\begin{array}{cc} \; & =\delta \left(\log\left(-\frac{f^{\left(n+1\right)}\left(\xi_{0}\right)}{(n+1)!}\right)+\left(n+1\right)\left(\log\left(\frac{\delta }{2}\right)-1\right)\right). \end{array}$$

For any δ>0, II is real and finite.

Our next goal is to show that the term III:=I−II is also finite for δ>0 small enough. We start by bounding $S:=\frac{f^{\left(n+2\right)}\left(\xi_{0}\right)}{(n+2)!}+\frac{f^{\left(n+3\right)}\left(\xi_{0}\right)}{(n+3)!}{\left(\xi -\xi_{0}\right)}^{1}+\ldots$:

(37) $$\begin{array}{cc} \left|S\right| & =\left|\frac{f^{\left(n+2\right)}\left(\xi_{0}\right)}{(n+2)!}+\frac{f^{\left(n+3\right)}\left(\xi_{0}\right)}{(n+3)!}{\left(\xi -\xi_{0}\right)}^{1}+\ldots \right|\\ \; & \leq \left|\frac{f^{\left(n+2\right)}\left(\xi_{0}\right)}{(n+2)!}\right|+\left|\frac{f^{\left(n+3\right)}\left(\xi_{0}\right)}{{}^{\left(n+3\right)}!}{\left(\xi -\xi_{0}\right)}^{1}\right|+\ldots \end{array}$$

The largest value for $|\xi -\xi_{0}|$ we have to consider is $|\xi -\xi_{0}|=\delta /2$. Hence,

(38) $$\begin{array}{cc} \left|S\right| & \leq \left|\frac{f^{\left(n+2\right)}\left(\xi_{0}\right)}{(n+2)!}\right|+\left|\frac{f^{\left(n+3\right)}\left(\xi_{0}\right)}{(n+3)!2}\right|\delta +\left|\frac{f^{\left(n+4\right)}\left(\xi_{0}\right)}{(n+4)!4}\right|\delta^{2}+\left|\ldots \right| \end{array}$$

Since, by assumption $\sup_{k=n+2,n+3,\ldots }\left|f^{\left(k\right)}\left(\xi_{0}\right)\right|<\infty$, we find that

(39) $$\begin{array}{cc} \left|S\right| & \leq \underset{k=n+2,n+3,\ldots }{\sup}\left|f^{\left(k\right)}\left(\xi_{0}\right)\right|\left(\frac{1}{(n+2)!}+\frac{\delta }{(n+3)!2}+\frac{\delta^{2}}{(n+4)!4}+\ldots \right)\\ \; & \to \underset{k=n+2,n+3,\ldots }{\sup}\left|f^{\left(k\right)}\left(\xi_{0}\right)\right|\frac{1}{(n+2)!}\;\mathrm{for}\;\delta \to 0, \end{array}$$

Hence,

(40) $$|S|<\frac{2}{(n+2)!}\underset{k=n+2,n+3,\ldots }{\sup}\left|f^{\left(k\right)}\left(\xi_{0}\right)\right|<\infty$$

for δ>0 small enough. The integral

(41) $$\begin{array}{cc} III & :=I-II \end{array}$$

(42) $$\begin{array}{cc} \; & =\int_{\xi_{0}-\delta /2}^{\xi_{0}+\delta /2}\log\left(1-f\left(\xi \right)\right)-\log\left(-\frac{f^{\left(n+1\right)}\left(\xi_{0}\right)}{(n+1)!}{\left(\xi -\xi_{0}\right)}^{\left(n+1\right)}\right)d\xi \end{array}$$

(43) $$\begin{array}{cc} \; & =\int_{\xi_{0}-\delta /2}^{\xi_{0}+\delta /2}\log\left(\frac{1-f(\xi )}{-\frac{f^{\left(n+1\right)}\left(\xi_{0}\right)}{(n+1)!}{\left(\xi -\xi_{0}\right)}^{\left(n+1\right)}}\right)d\xi \end{array}$$

(44) $$\begin{array}{cc} \; & =\int_{\xi_{0}-\delta /2}^{\xi_{0}+\delta /2}\log\left(\frac{-\frac{f^{\left(n+1\right)}\left(\xi_{0}\right)}{(n+1)!}{\left(\xi -\xi_{0}\right)}^{\left(n+1\right)}-\frac{f^{\left(n+2\right)}\left(\xi_{0}\right)}{(n+2)!}{\left(\xi -\xi_{0}\right)}^{\left(n+2\right)}+\ldots }{-\frac{f^{\left(n+1\right)}\left(\xi_{0}\right)}{(n+1)!}{\left(\xi -\xi_{0}\right)}^{\left(n+1\right)}}\right)d\xi \end{array}$$

(45) $$\begin{array}{cc} \; & =\int_{\xi_{0}-\delta /2}^{\xi_{0}+\delta /2}\log\left(1+\frac{\frac{f^{\left(n+2\right)}\left(\xi_{0}\right)}{(n+2)!}{\left(\xi -\xi_{0}\right)}^{\left(n+2\right)}+\ldots }{\frac{f^{\left(n+1\right)}\left(\xi_{0}\right)}{(n+1)!}{\left(\xi -\xi_{0}\right)}^{\left(n+1\right)}}\right)d\xi \end{array}$$

(46) $$\begin{array}{cc} \; & =\int_{\xi_{0}-\delta /2}^{\xi_{0}+\delta /2}\log\left(1+\left(\xi -\xi_{0}\right)\frac{(n+1)!}{f^{\left(n+1\right)}\left(\xi_{0}\right)}S\right)d\xi , \end{array}$$

is, in light of Equation (40), thus indeed finite for δ>0 small enough. Earlier, we already found that the integral II is finite for any δ>0. However, then, the integral I=II+III has to be finite as well for δ>0 small enough since the Lebesgue integrable functions form a vector space.

As f(ξ) is real-analytic on R, there can be only a finite number of points $\xi_{1}^{\circ },\xi_{2}^{\circ },\ldots ,\xi_{M}^{\circ }$ in [−W,W] at which $f(\xi_{m}^{\circ })=1$ [36] [Corollary 1.2.6]. (An infinite sequence $\xi_{1}^{\circ },\xi_{2}^{\circ },\cdots$ of spectral singularities in a finite interval [−W,W] would have an accumulation point. Similarly to before, this would imply $f\left(\xi \right)={\left|b\left(\xi \right)\right|}^{2}=1$ for all ξ, which contradicts Equation (26).) As shown above, we can choose $\delta_{1},\delta_{2},\ldots ,\delta_{M}>0$ small enough such that

(47) $$I_{m}:=\int_{\xi_{m}^{\circ }-\delta_{m}/2}^{\xi_{m}^{\circ }+\delta_{m}/2}\log\left(1-f\left(\xi \right)\right)d\xi >-\infty$$

for all *m*. The set

(48) $$X:=\left[-W,W\right]\setminus \overset{M}{\underset{m=1}{⋃}}\left(\xi_{m}^{\circ }-\delta_{m}/2,\xi_{m}^{\circ }+\delta_{m}/2\right)$$

is compact. The function f(ξ) thus attains a maximum on *X*, which has to be smaller than one since we removed all points where f(ξ)=1 from *X*. Summarizing, we find that

(49) $$\begin{array}{cc} E_{W} & =-\frac{1}{\pi }\left(\int_{X}\log\left(1-f\left(\xi \right)\right)d\xi +\int_{[-W,W]\setminus X}\log\left(1-f\left(\xi \right)\right)d\xi \right)\\ \; & \leq -\frac{1}{\pi }\left(\underset{>-\infty }{\underset{︸}{\min_{\xi \in X}\log\left(1-f\left(\xi \right)\right)}}\int_{X}d\xi +\sum_{m=1}^{M}I_{m}\right)<\infty . \end{array}$$

 □

Lemma 1 implies that there is a bound on the energy of b(ξ) when the nonlinear bandwidth is fixed. This leads us to the following lemma.

**Lemma** **2** (Energy bound for *b*-modulation)**.** *We consider the b-modulator in Theorem 2. Let 0<W<∞. Then, there exists a finite constant ${\bar{E}}_{W}$ such that the energy of any generated b(ξ) in [−W,W] satisfies*

(50) $$E_{W}=-\frac{1}{\pi }\int_{-W}^{W}{\log(1-|b\left(\xi \right)|}^{2})d\xi \leq {\bar{E}}_{W}.$$

The constant ${\bar{E}}_{W}$ depends on *Ψ*, $S_{*}$, N, Δξ, and W, but is independent of A and the choice of the $s_{n}$.

**Figure 5:**
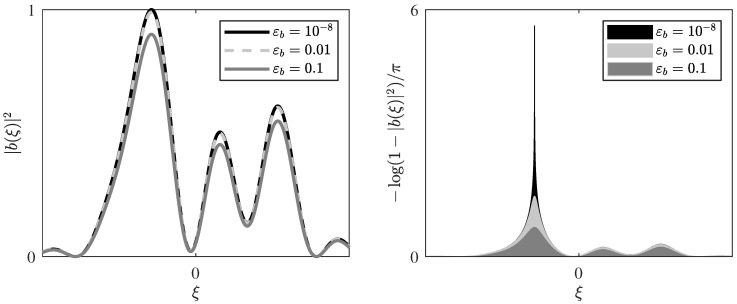
The left plot shows a b(ξ) of the form Equation (20) for several values of the power control factor *A*, resulting in different gaps to singularity $\varepsilon_{b}=1-{∥b∥}_{\infty }^{2}$. The right plot shows the corresponding integrand in Equation (50). The shaded areas thus represent the signal energy $E_{W}$ in the shown interval. Lemma 2 tells us that $E_{W}$ will stay below a finite bound no matter how small the gap to singularity becomes.

Figure 5 presents a graphical illustration of Lemma 2.

**Proof of Lemma** **2.** Let us fix $s_{-N},\ldots ,s_{N}\in S_{*}$. For a real-analytic Ψ(ξ), $s_{n}\Psi \left(\xi \right)$ will also be real-analytic [36] [Proposition 1.1.4]. The sum of real-analytic functions is also real-analytic [36] [Proposition 1.1.4], thus b(ξ) will be real-analytic. Since *A* is assumed admissible, the first condition in Equation (26) is fulfilled. The first assumption in Equation (21) ensures that the second condition in Equation (26) is fulfilled as well. By applying the triangle inequality to Equation (26) and using Equation (21) to bound the individual summands, we find that also the third condition in Equation (26) is fulfilled. Hence, we can apply Lemma 1. The admissible *A* that results in the largest energy in [−W,W] is given by (We assume without loss of generality that the denominator in Equation (51) is not zero. In such cases, the energy is zero for all A≥0.)

(51) $$A_{*}=1/\max_{\xi \in R}\left|\sum_{n=-N}^{N}s_{n}\Psi \left(\xi -n\Delta \xi \right)\right|.$$

Lemma 1 shows that $E_{W}$ is finite for the choice $A=A_{*}$. Since $E_{W}$ can only be lower for other admissible choices of *A*, we have obtained a finite upper bound on $E_{W}$ for the chosen $s_{-N},\ldots ,s_{N}$ that is independent of *A*. Since our constellation alphabet is finite, there is only a finite number of choices for the $s_{-N},\ldots ,s_{N}$. By taking the maximum over the upper bounds on $E_{W}$ for each possible choice, we obtain an upper bound on $E_{W}$ that is independent of both *A* and the $s_{n}$. □

Now that we have proved the existence of an energy bound for the modulator in Theorem 2, we shall proceed to prove the power bound.

**Proof of Theorem** **2.** Our first goal is to bound the energy corresponding to the nonlinear spectrum

$$b\left(\xi \right)=Ab_{0}\left(\xi \right):=A\sum_{n=-N}^{N}s_{n}\Psi \left(\xi -n\Delta \xi \right).$$

As the energy is always zero if $∥b_{0}{∥}_{\infty }=0$, we assume without loss of generality that $∥b_{0}{∥}_{\infty }>0$. Let us fix an arbitrary $0<\delta <∥b_{0}{∥}_{\infty }$. Since Ψ(ξ)→0 for ξ→±∞, also $b_{0}\left(\xi \right)\to 0$ for ξ→±∞. Hence, we can choose 0<W<∞ such that $|b_{0}\left(\xi \right)|<\delta$ for all |ξ|>W. Since $A∥b_{0}{∥}_{\infty }\leq 1$ by Equation (14), we obtain

$$A\delta \leq \frac{\delta }{∥b_{0}{∥}_{\infty }}=:\eta <1$$

for any admissible A≥0. Choose now ${\bar{E}}_{W}$ as in Lemma 2. Then,

(52) $$\begin{array}{cc} E & =-\frac{1}{\pi }\int_{-\infty }^{\infty }\log(1-A^{2}|b_{0}{|}^{2})d\xi \end{array}$$

(53) $$\begin{array}{cc} \; & =-\frac{1}{\pi }\int_{R\setminus [-W,W]}\log(1-A^{2}|b_{0}{|}^{2})d\xi -\frac{1}{\pi }\int_{-W}^{W}\log(1-A^{2}|b_{0}{|}^{2})d\xi \end{array}$$

(54) $$\begin{array}{cc} \; & \leq -\frac{1}{\pi }\int_{R\setminus [-W,W]}\log(1-A^{2}|b_{0}{|}^{2})d\xi +{\bar{E}}_{W} \end{array}$$

(55) $$\begin{array}{cc} \; & =\frac{1}{\pi }\int_{R\setminus [-W,W]}\left(A^{2}|b_{0}{\left(\xi \right)|}^{2}+\frac{1}{2}A^{4}|b_{0}{\left(\xi \right)|}^{4}+\frac{1}{3}A^{6}{\left|b_{0}\left(\xi \right)\right|}^{6}+\cdots \right)d\xi +{\bar{E}}_{W} \end{array}$$

(56) $$\begin{array}{cc} \; & \leq \frac{1}{\pi }\int_{R\setminus [-W,W]}A^{2}{\left|b_{0}\left(\xi \right)\right|}^{2}\left(1+\frac{1}{2}A^{2}\delta^{2}+\frac{1}{3}A^{4}\delta^{4}+\cdots \right)d\xi +{\bar{E}}_{W} \end{array}$$

(57) $$\begin{array}{cc} \; & \leq \frac{1}{\pi }\frac{\eta^{2}}{\delta^{2}}\left(1+\eta^{2}+\eta^{4}+\cdots \right)\int_{R\setminus [-W,W]}{\left|b_{0}\left(\xi \right)\right|}^{2}d\xi +{\bar{E}}_{W} \end{array}$$

(58) $$\begin{array}{cc} \; & \leq \frac{1}{\pi }\frac{\eta^{2}}{\delta^{2}}\left(1+\eta^{2}+\eta^{4}+\cdots \right){∥b_{0}∥}_{2}^{2}+{\bar{E}}_{W}\;<\;\infty . \end{array}$$

In Equation (52), the Taylor expansion $-\log\left(1-\xi^{2}\right)=\xi^{2}+\xi^{4}/2+\xi^{6}/3+\cdots$ was used. In Equation (), it was used that Aδ≤η. In the last line, we used $∥b_{0}{∥}_{2}^{2}<\infty$, which follows from $\Psi \in L^{2}\left(R\right)$, and 0<η<1.

The bound on *E* in Equation () is independent of *A* but still depends on the choice of the $s_{-N},\cdots ,s_{N}\in S_{*}$ used to construct $b_{0}\left(\xi \right)$. Since the set $S_{*}$ is finite, there is only a finite number of possible $b_{0}\left(\xi \right)$. Let $\bar{E}$ denote the largest value of Equation (58) over all possible $b_{0}\left(\xi \right)$. By construction, $\bar{E}$ is finite and independent of both *A* and the choice of $s_{-N},\cdots ,s_{N}$. From Equation (14), we find $\int_{T_{1}}^{T_{2}}{\left|q\left(t\right)\right|}^{2}dt\leq \int_{-\infty }^{\infty }{\left|q\left(t\right)\right|}^{2}dt=E\leq \bar{E}$. Thus, $P\leq \bar{E}/\left(T_{2}-T_{1}\right)$ with $\bar{E}$ independent of *A*, the choice of the $s_{n}$, and of course the duration $T_{2}-T_{1}$. □

## 4. Conclusions

The NFDM technique of *b*-modulation has received much attention in the last few years. We have shown, for the first time, that, for *b*-modulators, the nonlinear bandwidth, signal duration, and power are coupled when, as it is the case in most practical implementations, the gap to singularity is bounded. For fixed nonlinear bandwidth, this results in a bound on the transmit power that decreases with signal duration. This decrease in the transmit power implies that the supremum of the achievable signal-to-noise ratios (SNRs) decreases as the signals become longer. Hence, we established a new factor that contributes to the observed performance degradation of *b*-modulated systems for long signals [10,11]. Furthermore, we showed that, even in the presence of spectral singularities, the transmit powers of many *b*-modulators cannot be made arbitrarily large. The results in this paper also apply to NFDM systems that modulate the reflection coefficient in fibers with normal dispersion when *b* is replaced with ρ since the underlying mathematical structure is the same. The cases of *b*-modulation in normal dispersion fiber and ρ-modulation in anomalous dispersion fiber require further research.

## Abbreviations

The following abbreviations are used in this manuscript:

NFT	Nonlinear Fourier Transform
NFDM	Nonlinear Frequency Division Multiplexing
